# Essential Oils from Leaves of Medicinal Plants of Brazilian Flora: Chemical Composition and Activity against *Candida* Species

**DOI:** 10.3390/medicines4020027

**Published:** 2017-05-03

**Authors:** Maria da Conceição Mendes Ferreira da Costa, Alexandre Gomes da Silva, Ana Paula Sant’Anna da Silva, Vera Lúcia de Menezes Lima, Patrícia Cristina Bezerra-Silva, Suyana Karolyne Lino da Rocha, Daniela Maria do Amaral Ferraz Navarro, Maria Tereza dos Santos Correia, Thiago Henrique Napoleão, Márcia Vanusa da Silva, Patrícia Maria Guedes Paiva

**Affiliations:** 1 Departamento de Bioquímica, Centro de Biociências, Universidade Federal de Pernambuco, Cidade Universitária, Recife 50670-420, Pernambuco, Brazil; ceacosta.ufpe@gmail.com (M.C.M.F.C.); annapsantanna@hotmail.com (A.P.S.S.); vlml@ufpe.br (V.L.M.L.); mtscorreia@gmail.com (M.T.S.C.); thiagohn86@yahoo.com.br (T.H.N.); marciavanusa@yahoo.com.br (M.V.S.); 2 Instituto Nacional do Semiárido, Campina Grande 58429-970, Paraíba, Brazil; agsilva@live.com; 3 Departamento de Química Fundamental, Centro de Ciências Exatas e da Natureza, Universidade Federal de Pernambuco, Recife 50670-901, Pernambuco, Brazil; patricia.c.bezerra@hotmail.com (P.C.B.-S.); suyanarocha@hotmail.com (S.K.L.R.); navarrix@uol.com.br (D.M.A.F.N.)

**Keywords:** essential oils, antifungal activity, Brazilian flora, Fabaceae, Hypericaceae

## Abstract

**Background**: The biotechnological potential of medicinal plants from Brazilian Caatinga and the Atlantic Forest has not been extensively studied. Thus, screening programs are important in prospecting for compounds for developing new drugs. The purpose of this study was to determine the chemical composition and to evaluate the anti-*Candida* activity of essential oils from leaves of *Hymenaea courbaril* var. *courbaril*, *Myroxylon peruiferum*, and *Vismia guianensis*. **Methods**: The oils were extracted through hydrodistillation and their chemical compositions were analyzed by gas chromatography coupled with mass spectrometry. Antifungal activity against *C. albicans*, *C. tropicalis*, *C. parapsilosis*, *C. glabrata*, and *C. krusei* was evaluated by determining the minimal inhibitory (MIC) and fungicidal (MFC) concentrations. **Results**: The major compounds of the oils were caryophyllene oxide and *trans*-caryophyllene for *H. courbaril*; spathulenol, α-pinene, and caryophyllene oxide for *M. peruiferum*; and caryophyllene oxide and humulene epoxide II for *V. guianensis* oil. The oils showed antifungal activity against all the strains tested, and the MIC values ranged between 0.625 and 1.25 μL/mL and MFC from 0.625 to 2.5 μL/mL. **Conclusion**: The essential oils from the species studied have the potential to be evaluated as clinical applications in the treatment of candidiasis.

## 1. Introduction

Candidiasis is the most frequent infection caused by opportunistic fungi, with a very extensive spectrum ranging from mild manifestations (e.g., a colonization of mucosal tissues) to systemic infections with the invasion of different organs [1]. *Candida albicans* is the most common causative agent in humans, but the species *Candida tropicalis*, *Candida parapsilosis*, *Candida glabrata*, and *Candida krusei* are also commonly associated with serious infections [2,3,4]. In addition, *C. glabrata* and *C. krusei* are described as having intrinsic resistance to some of the more conventional antifungal drugs [5,6].

Azole antifungal agents are the most used in therapeutic regimens for the treatment of candidiasis. However, these antibiotics are not always successful because of the significant incidence of microorganism resistance [7]. This has stimulated the search for new antifungal agents in order to increase the number of alternatives to treat infections and minimize the impact of resistance.

Natural products have been traditionally used to treat many diseases, including fungal infections, and plant compounds have been explored by the pharmaceutical industry for use as antimicrobial agents or as models for the synthesis of new drugs [8]. Essential oils, which consist in liquid, are volatile, and are rarely colored mixtures of compounds soluble in organic solvents, are among these potential alternatives [9,10]. The essential oils are usually constituted by some components at high concentrations (20–70%), being deemed majoritary compounds, while the other components are present in trace amounts [11].

Plants from Caatinga and Atlantic Forest have also been used to obtain natural medicines by Brazilian local populations [12]. However, despite the rich flora of these biomes, the biotechnological potential of plants as sources of antifungal agents has not been extensively studied. Thus, screening programs are very important in order to prospect for compounds for developing new antimicrobial drugs. 

This study was conducted with essential oils from three medicinal plants of Brazilian flora. *Hymenaea courbaril* var. *courbaril* (Fabaceae) has a broad distribution being found in Brazil at Atlantic and Amazon Forests, Caatinga, Cerrado, and Pantanal. It has several medicinal uses; for example, the stem bark decoction or syrup can be used against coughs and anemia, and leaf infusion can be used against urinary tract disorders [13,14]. *Myroxylon peruiferum* (Fabaceae) is found at the Brazilian Atlantic Forest and Cerrado and has heavy wood that is resistant to deterioration. Its bark is used as an anti-inflammatory, and the fruits are used to alleviate earache [15,16]. The leaves of *Vismia guianensis* (Hypericaceae) are used against problems in spine and kidneys as well as to alleviate pains in general [17]. This plant can be found in the Amazon and Atlantic Forests [13].

The aim of this work was to determine the chemical composition of essential oils from leaves of *H. courbaril* var. *courbaril*, *M. peruiferum*, and *V. guianensis* and to evaluate the antifungal activity of them against *Candida* species.

## 2. Materials and Methods 

### 2.1. Plant Materials

Leaves of *V. guianensis* were collected in December 2012, at the nature reserve of Camaçari (8°16′23′′ S, 34°57′45′′ W) at Cabo de Santo Agostinho, Pernambuco, Brazil, at an Atlantic Forest region. Leaves of *H. courbaril* var. *courbaril* and *M. peruiferum* were collected in March 2013 at the Vale do Catimbau National Park (08°30′02.3′′ S, 37°20′31′′ W) in Pernambuco, Brazil, at a Caatinga region. For each plant, leaves were collected from at least 15 individuals and pooled for oil extraction. Plant collection was performed with authorization (number 16806) of the *Instituto Chico Mendes de Conservação da Biodiversidade* (ICMBio). Voucher specimens of *H. courbaril* var. *courbaril* (number 84888), *M. peruiferum* (number 84110), and *V. guianensis* (number 90471) are deposited in the herbarium “Dárdano de Andrade Lima” from the *Instituto Agronômico de Pernambuco*, Recife, Brazil. The leaves were dried at 28 °C, milled to a fine powder, and used immediately for the extraction of oils. 

### 2.2. Extraction of Essential Oils

The powders of leaves were separately submitted to hydrodistillation in a Clevenger-type apparatus for 4 h. The essential oils obtained were then dried over an anhydrous sodium sulfate and stored in sealed vials protected from the light at −20 °C. The essential oil emulsions used in antifungal assays were prepared as follows: 400 µL of essential oil plus 40 µL of Tween 80 and 4.56 mL of sterile distilled water were combined and shaken for 5 min using a vortex, obtaining emulsions with a final concentration of 80 μL/mL. 

### 2.3. Chromatography Analyses

Gas chromatography (GC) analyses were performed in order to determine the relative proportions of the components of the oils. GC analyses were carried on a Thermo TraceGC Ultra (Thermo Scientific, Milan, Italy) equipped with a flame ionization detector (FID) (Thermo Scientific, Milan, Italy) and a VB-5 fused silica capillary column (ValcoBond 30 m × 0.25 mm i.d.; film thickness: 0.25 mm) (Valco Instruments Company Inc. , Houston, TX, USA). Nitrogen was employed as a carrier gas at a flow rate of 1 L/min and 30 psi inlet pressure. The oven temperature program was initially 40 °C, was held for 2 min, was increased to 230 °C at 4 °C/min, and was then held for 5 min. The injector and detector temperatures were set to 250 °C and 280 °C, respectively. The sample (1 µL; 2 mg/mL in n-hexane) was injected splitless. The relative amount of each component was estimated from the corresponding peak area and expressed as a percentage of the total area of the chromatogram. Analyses were carried out in triplicate.

The identification of the compounds present in the oils was performed by GC coupled with mass spectrometry (GC-MS). These analyses were carried out using an Agilent 5975C Series GC/MSD (Agilent Technologies, Palo Alto, CA, USA) quadrupole instrument equipped with an Agilent J&W non-polar DB-5 fused silica capillary column (30 m × 0.25 mm i.d.; film thickness: 0.25 μm) (Agilent Technologies, Palo Alto, CA, USA). For each sample, 1 μL was injected in split mode (50:1) with the injector temperature set to 250 °C. GC oven temperature was set to 40 °C, was held for 2 min, was increased to 230 °C at 4 °C/min, and was then held for 5 min. Helium (He) was used as a carrier gas at a flow of 1 mL/min, maintained at a constant pressure of 7.0 psi. MS Source and quadrupole temperatures were set to 230 °C and 150 °C, respectively. Mass spectra were taken at 70 eV (in EI mode) with a scanning speed of 1.0 scans from *m*/*z* 35–350. The identification of the individual components was carried out by comparison with previously reported values of retention indices, obtained by co-injection of oil samples and a set of C_9_–C_30_ linear hydrocarbons, and calculated according to the equation of Van den Dool and Kratz [18]. Subsequently, the MS data acquired for each component were matched with the data available in the mass spectral library of the GC-MS system (MassFinder 4, Dr. Hochmuth scientific consulting, Hamburg, Germany); NIST08 Mass Spectral Library (ChemSW Inc. Fairfield, CA, USA); Wiley Registry™ of Mass Spectral Data 9th Edition (Wiley, Hoboken, NJ, USA) and with other published mass spectral data [19]. Analyses were carried out in triplicate. 

### 2.4. Antifungal Assays

Strains of *C. tropicalis* (URM-6741), *C. krusei* (URM-6391), *C. albicans* (URM-6543), *C. parapsilosis* (URM-6557), and *C. glabrata* (URM-6393) were obtained from the University Recife Mycologia (URM) from the *Departamento de Micologia* of *Universidade Federal de Pernambuco*. The yeasts were grown overnight at 30 °C in Petri plates containing Sabouraud dextrose agar (Merck, Darmstadt, Germany), and inocula for use in the assays were prepared by diluting scraped cell mass in 0.85% NaCl, adjusting to 0.5 in McFarland. Cell suspensions were finally diluted to 10^6^ CFU/mL for use in the assays. 

The minimal inhibitory concentrations (MICs) of the essential oils were determined through the microdilution technique. Firstly, 100 µL of Sabouraud dextrose broth (Merck, Darmstadt, Germany) were taken into the wells of a 96-well microtiter plate. The first well of each row was used as a sterility control and contained only culture medium. Next, 100 µL of the emulsion of oil emulsion (80 μL/mL) were placed in the second well of a row and two-fold serial dilution was then performed until a concentration of 0.04 μL/mL was reached. In each well, aliquots of 10 µL of the microorganism inoculum were dispensed. In parallel, a negative control containing only microorganism and medium was run to assure the viability of the yeast strains. It was also performed a positive control using the antifungal fluconazole (Sigma-Aldrich, St. Louis, MO, USA). Assays were performed in triplicate and plates were incubated at 30 °C for 48 h. After this period, 10 µL of the dye 2,3,5-triphenyl tetrazolium chloride (0.5%, *w*/*v*) was added in each well to detect bacterial growth. The MIC was defined as the lowest oil concentration that promoted inhibition of yeast growth. 

After the determination of the MIC, aliquots from the wells where there was growth inhibition, as well as from positive and negative controls, were subcultured on Petri plates containing Sabouraud dextrose agar (Merck). After 24 h at 30 °C, the microbial growth was observed and the number of CFU was counted. The minimal fungicide concentration (MFC) was defined as the lowest oil concentration that reduced the number of CFU in 99.9% in comparison with the initial inoculum. 

According to the MFC/MIC ratio, the antifungal activity was classified as follows: if MFC/MIC = 1 or 2, the effect was considered fungicidal; if MFC/MIC ≥ 4, the effect was defined as fungistatic.

## 3. Results

The yields of oil extraction were 0.975%, 0.276%, and 0.306% for *H. courbaril*, *M. peruiferum*, and *V. guianensis*, respectively. The chemical composition of the essential oils can be seen in Table 1. A total of 62 compounds were identified in the essential oils. The oils of the species *H. courbaril*, *M. peruiferum*, and *V. guianensis* were rich in mono and sesquiterpenes. The *trans*-caryophyllene, δ-cadinene and caryophyllene oxide were present in all the three oils. 

All tested *Candida* species were sensitive to the essential oils and MIC values ranged from 0.625 to 1.25 μL/mL (Table 2). *C. albicans* and *C. krusei* were more sensitive to the *V. guianensis* oil, while *C. glabrata* and *C. parapsilosis* were more sensitive to the *H. courbaril* oil. All the oils showed fungicidal activity and all *Candida* strains were sensitive to the positive control fluconazole (Table 2). According to MFC/MIC ratios, the effect of all oils on the *Candida* species can be considered fungicidal.

## 4. Discussion

As mentioned before, mono and sesquiterpenes were the main compounds identified in the materials used in the present work, which is commonly reported for essential oils [21]. Andrade et al. [22] evaluated an essential oil from *M. peruiferum* bark and found a chemical composition highly distinct from that found for the leaf oil. None of the majoritary compounds of the leaf oil was present in the bark oil, and two of the major compounds of the bark oil (α-copaene and δ-Cadinene) were detected in the leaf oil but in small concentrations. A similar situation is observed when comparing the composition of an essential oil from *H. courbaril* ripe fruit peel [23], and the leaf oil of *H. courbaril* var. courbaril that we described. The main components of the fruit oil were α-copaene, spathulenol, and β-selinene (11.1%, 10.1%, and 8.2%, respectively), which were only present in small concentrations in the leaf oil (0.41%–1.99%). 

All essential oils showed antifungal activity. The concentration range at which the essential oils were active was close to that reported for the essential oil of *Myrtus communis* leaves against *Candida* species [24]. The mechanisms of antifungal activity of essential oils seem to involve the disruption of cell membrane structure causing leakage and cell death, blocking of membrane synthesis, and cellular respiration as well as inhibition of the spore germination [25]. An essential oil from *Coriandrum sativum* leaves, containing the mono- and sesquiterpene hydrocarbons decanal, trans-2-decenal, 2-decen-1-ol, and cyclodecane as major compounds, was active against *C. albicans* probably by binding to the membrane ergosterol, by increasing ionic permeability, and by decreasing proteolytic activity [26]. The authors also demonstrated that this oil did not interfere with cell wall biosynthesis pathways and was most active than isolated fractions, which suggest a synergistic effect between the oil components.

The antifungal activity of *H. courbaril* var. courbaril, *M. peruiferum*, and *V. guianensis* oils can be linked to the major compounds caryophyllene oxide and *trans*-caryophyllene. Caryophyllene oxide was proven to be an antifungal agent against *C. albicans* [27], and Vieira et al. [28] showed that essential oil from *Piper diospyrifolium*, rich in *trans*-caryophyllene, was active against clinical isolates of *C. albicans* and *C. parapsilosis*. Caryophyllene oxide also showed antifungal activity against dermatophytes compared to ciclopirox olamine and sulconazole [29]. 

Antifungal activity has been also reported for oils containing other compounds found in the oils analyzed here. Spathulenol and bicyclogermacrene (both found in *M. peruiferum* oil) were the main constituents of the essential oil from leaves of *Croton argyrophylloides*, which was active against *C. albicans*, *Candida tropicalis*, and *Microsporum canis* [30]. An essential oil from the rhizome of *Ferula hermonis* containing mainly α-pinene (also present in *M. peruiferum* oil) was active against *Candida albicans*, *Candida lactis-condensi*, *Saccharomyces cerevisiae*, *Tricophyton mentagrophytes*, *Microsporum gypseum*, *Aspergillus niger*, *Aspergillus fumigates*, and *Penicillium purpurogenum* [31]. The authors also reported that the compounds α-pinene and spathulenol were active against *C. albicans* when tested separately. 

## 5. Conclusions

The essential oils from three plants commonly used in Brazilian folk medicine were effective antifungal agents against *Candida* species. The antifungal activity of the oils is probably linked to the action of compounds such as caryophyllene oxide and *trans*-caryophyllene.

## Figures and Tables

**Table 1:** Identification of constituents of the essential oil from leaves of *Hymenaea courbaril* var. *courbaril* (HC), *Myroxylon peruiferum* (MP), and *Vismia guianensis* (VG).

Compound ^a^	Retention Index		Content (as % of Total Oil) + SD		
	Determined ^b^	Literature ^c^	HC	MP	VG
α-Pinene	932	932	-	9.64 ± 0.20	-
Thuja-2,4 (10)-diene	952	953	-	0.23 ± 0.01	-
β-Pinene	974	974	-	0.44 ± 0.01	-
Myrcene	991	988	-	0.15 ± 0.03	-
ρ-Cymene	1024	1020	-	0.10 ± 0.04	-
Limonene	1028	1024	-	7.17 ± 0.38	-
γ-Terpinene	1058	1054	-	0.12 ± 0.07	-
Terpinolene	1088	1086	-	0.05 ± 0.02	-
α-Campholenal	1126	1122	-	0.95 ± 0.04	-
*trans*-Pinocarveol	1138	1135	-	0.38 ± 0.06	-
*trans*-Verbenol	1144	1140	-	1.17 ± 0.15	-
Citronellal	1154	1148	-	-	0.19 ± 0.01
Pinocarvone	1163	1160	-	0.08 ± 0.05	-
α-Phellandre-8-ol	1167	1166	-	0.20 ± 0.00	-
Terpinen-4-ol	1177	1174	-	0.41 ± 0.01	-
*trans*-Carveol	1219	1215	-	0.22 ± 0.01	-
Isovaleric acid	1234	1232	-	0.22 ± 0.01	-
α-Cubebene	1351	1348	0.72 ± 0.08	1.19 ± 0.01	0.23 ± 0.02
α-Ylangene	1373	1373	-	-	0.18 ± 0.01
α-Copaene	1377	1374	1.95 ± 0.16	2.63 ± 0.11	3.35 ± 0.17
β-Bourbonene	1387	1387	-	4.96 ± 0.21	-
β-Cubebene	1392	1387	-	0.66 ± 0.02	-
β-Elemene	1393	1389	1.49 ± 0.07	6.79 ± 0.20	0.34 ± 0.06
Cyperene	1401	1398	2.28 ± 0.20	-	-
Ylanga-2,4 (15)-diene	1407	1400^d^	-	0.55 ± 0.03	-
*trans*-Caryophyllene	1421	1417	18.80 ± 0.10	3.51 ± 0.11	3.18 ± 0.17
β-Copaene	1431	1430	0.56 ± 0.08	-	0.54 ± 0.04
*trans*-α-Bergamotene	1437	1432	0.60 ± 0.09	-	-
Aromadendrene	1441	1439	0.27 ± 0.04	-	-
α-Humulene	1456	1452	2.89 ± 0.06	1.19 ± 0.06	1.09 ± 0.06
Caryophyllene<9-epi-(E)->	1464	1464	-	0.22 ± 0.00	-
γ-Muurolene	1479	1478	0.51 ± 0.06	0.42 ± 0.02	2.98 ± 0.35
Germacrene D	1486	1484	2.12 ± 0.16	1.06 ± 0.04	-
β-Selinene	1489	1489	1.99 ± 0.33	1.63 ± 0.04	0.62 ± 0.04
Viridiflorene	1497	1496	-	-	0.33 ± 0.02
α-Selinene	1498	1498	3.12 ± 0.32	1.22 ± 0.03	-
Bicyclogermacrene	1499	1500	-	5.49 ± 0.20	-
*trans*-β-Guaiene	1503	1502	1.20 ± 0.08	-	-
α-Muurolene	1503	1500	-	4.23 ± 0.50	0.55 ± 0.04
Germacrene A	1508	1508	-	2.67 ± 0.08	-
γ-Cadinene	1517	1513	0.63 ± 0.18	0.83 ± 0.02	2.03 ± 0.118
δ-Cadinene	1525	1522	1.16 ± 0.10	2.84 ± 0.02	0.96 ± 0.08
*trans*-Cadina-1,4-diene	1535	1533	-	0.18 ± 0.02	-
α-Cadinene	1540	1537	-	0.13 ± 0.08	0.31 ± 0.05
Unidentified Sesquiterpene	1544	-	-	-	0.86 ± 0.05
α-Calacorene	1546	1544	2.90 ± 0.05	0.37 ± 0.01	-
Unidentified Sesquiterpene	1555	-	0.23 ± 0.05	0.79 ± 0.01	2.54 ± 0.11
Unidentified compound	1562	-	-	-	0.61 ± 0.02
*trans*-Nerolidol	1565	1561	1.09 ± 0.18	-	0.64 ± 0.04
Unidentified Sesquiterpene	1569	-	1.38 ± 0.11	-	-
Spathulenol	1581	1577	0.41 ± 0.06	13.29 ± 0.48	0.74 ± 0.03
Caryophyllene oxide	1586	1582	29.55 ± 1.32	7.27 ± 0.27	46.8 ± 1.75
Salvial-4 (14)-em-1-one	1597	1594	0.46 ± 0.02	0.96 ± 0.05	1.12 ± 0.02
Unidentified Sesquiterpene	1602	-	-	-	0.94 ± 0.06
Unidentified Sesquiterpene	1607	-	-	-	0.55 ± 0.10
Humulene epoxide II	1612	1608	2.80 ± 0.12	-	7.21 ± 0.05
Unidentified Sesquiterpene	1628	-	15.62 ± 0.32	-	0.19 ± 0.11
Cubenol<1-epi->	1631	1627	0.51 ± 0.09	0.15 ± 0.01	1.73 ± 0.18
Unidentified Sesquiterpene	1635	-	-	-	1.45 ± 0.18
Caryophylla-4 (12),8 (13)-dien-5-ol	1639	1639	0.34 ± 0.05	-	0.84 ± 0.40
τ-Muurolol	1645	1640	0.50 ± 0.20	0.12 ± 0.04	0.6 ± 0.16
α-Cadinol	1657	1652	2.00 ± 0.08	-	0.93 ± 0.33
Pogostol	1660	1651	-	-	1.73 ± 0.10
*trans*-Calamenen-10-ol	1671	1668	-	-	4.58 ± 0.52
Caryophyllene<14-hydroxy-9-epi (E)->	1673	1668	0.36 ± 0.06	-	-
Cadelene	1678	1675	-	-	0.54 ± 0.07
Muskatone	1681	1676	-	-	0.37 ± 0.11
Unidentified compound	1685	-	-	-	0.75 ± 0.17
Eudesma-4 (15),7-dien-1β-ol	1689	1687	0.21 ± 0.08	0.33 ± 0.27	-
Unidentified Sesquiterpene	1694	-	-	0.16 ± 0.08	-
Unidentified Sesquiterpene	1707	-	-	-	0.28 ±0.06
Unidentified Sesquiterpene	1711	-	-	0.66 ± 0.13	-
Unidentified Sesquiterpene	1720	-	-	-	0.22 ± 0.01
Unidentified Sesquiterpene	1721	-	-	0.49 ± 0.13	-
Unidentified Sesquiterpene	1728	-	-	-	0.51 ± 0.06
Muurolene<14-oxy- α>	1769	1767	-	-	0.52 ± 0.06
Unidentified Sesquiterpene	1774	-	-	-	0.19 ± 0.06
Methylhexadecanoate	1926	1921	-	-	0.87 ± 0.12
Unidentified compound	1968	-	0.63 ± 0.10	-	-
Total			99.3	98.36	95.19

^a^ Constituents listed in order of elution on a non-polar DB-5 column. ^b^ Retention indices (RI) calculated from retention times in relation to those of a series of C_9_-C_30_*n*-alkanes on a 30 m DB-5 capillary column. ^c^ Values taken from Adams [19]. ^d^ Value taken from Bos et al. [20].

**Table 2:** Minimum inhibitory concentrations (MICs) and minimal fungicidal concentrations (MFCs) on *Candida* species of the essential oils from leaves of *H. courbaril* var. *courbaril*, *M. peruiferum*, and *V. guianensis* as well as of positive control fluconazole.

Sample		Fungi				
		*C. albicans*	*C. glabrata*	*C. krusei*	*C. parapsilosis*	*C. tropicalis*
*H. courbaril* var. *courbaril*	MIC (µL/mL)	1.25	0.625	1.25	1.25	0.625
	MFC (µL/mL)	2.5	1.25	1.25	2.5	0.625
	MFC/MIC	2	2	1	2	1
*M. peruiferum*	MIC (µL/mL)	1.25	1.25	0.625	0.625	1.25
	MFC (µL/mL)	2.5	1.25	1.25	1.25	2.5
	MFC/MIC	2	1	2	2	2
*V. guianensis*	MIC (µL/mL)	0.625	1.25	0.625	1.25	1.25
	MFC (µL/mL)	1.25	2.5	1.25	2.5	2.5
	MFC/MIC	2	2	2	2	2
Fluconazole	MIC (µg/mL)	1.56	1.56	0.39	0.78	1.56
	MFC (µg/mL)	3.12	1.56	0.39	1.56	1.56
	MFC/MIC	2	1	1	2	1
